# Functional Identification of Complement Factor D and Analysis of Its Expression during GCRV Infection in Grass Carp (*Ctenopharyngodon* *idella*)

**DOI:** 10.3390/ijms222112011

**Published:** 2021-11-06

**Authors:** Chunhua Ding, Tiaoyi Xiao, Beibei Qin, Baohong Xu, Zhao Lv, Hongquan Wang

**Affiliations:** Hunan Engineering Technology Research Center of Featured Aquatic Resources Utilization, Hunan Agricultural University, Changsha 410128, China; 15251125135@163.com (C.D.); tiaoyixiao@hunau.edu.cn (T.X.); qinbeibei0@gmail.com (B.Q.); xbh_1012@hunau.edu.cn (B.X.)

**Keywords:** complement factor D, *Ctenopharyngodon idella*, cleavage of C3, GCRV, expression patterns

## Abstract

Complement factor D (Df) is a serine protease well known for activating the alternative pathway (AP) in mammals by promoting the cleavage of complement component 3 (C3), thus becoming involved in innate defense. In teleost fish, however, the functional mechanisms of Df in the AP and against pathogen infection are far from clear. In the present study, we cloned and characterized the Df gene, *Ci*Df, from grass carp (*Ctenopharyngodon idella*) and analyzed its function in promoting C3 cleavage and expression changes after grass carp reovirus (GCRV) infection. The open reading frame of *Ci*Df was found to be 753 bp, encoding 250 amino acids with a molecular mass of 27.06 kDa. *Ci*Df harbors a conserved Tryp_SPc domain, with three conserved residues representing the catalytic triad and three conserved binding sites in the substrate specificity pocket. Pairwise alignment showed that *Ci*Df shares the highest identity (96%) and similarity (98%) with Df from *Anabarilius grahami*. Phylogenetic analysis indicated that *Ci*Df and other fish Dfs formed a distinct evolutionary branch. Similar to most Dfs from other vertebrates, the *Ci*Df gene structure is characterized by four introns and five exons. The incubation of recombinant *Ci*Df protein with grass carp serum significantly increased the C3b content, demonstrating the conserved function of *Ci*Df in the AP in promoting C3 cleavage, similar to Dfs in mammals. *Ci*Df mRNA expression was widely detected in various tissues and levels were relatively higher in the liver, spleen, and intestine of grass carp. During GCRV infection over a 168-hour period, a high level of *Ci*Df mRNA expression in the liver, spleen, and intestine was maintained at 144 and 168 h, suggesting AP activity at the late stage of GCRV infection. Collectively, the above results reveal the conserved structure and function of *Ci*Df and its distinct expression patterns after GCRV infection, which provide a key basis for studying the roles of Df and AP during GCRV infection in the grass carp *C. idella*.

## 1. Introduction

The complement system is considered one of the most important components of the innate immune system. It plays a pivotal role in the immune defense of animal hosts against pathogens, including bacteria and viruses [1,2]. This system is composed of more than 35 secreted serum proteins and membrane-bound proteins that undergo cascade hydrolysis or activation upon microbial pathogen invasion, ultimately triggering the activation of the complement system to eliminate these pathogens [1,2,3]. It has generally been accepted that there are three pathways of complement system activation: the classical pathway (CP), the lectin pathway (LP), and the alternative pathway (AP) [1,2,4]. The CP is activated by the binding of a plasma protein called C1 (the first component of the complement system) to antibodies (IgG or IgM) bound to the surface of a microbe [5]; the LP is initiated by a plasma protein called mannose-binding lectin, which identifies terminal mannose residues on microbial glycoproteins and glycolipids, or by ficolin, which displays lectin activity and is usually specific for *N*-acetylglucosamine [6], and the AP is triggered by the direct recognition of certain microbial surface structures [7]. All three pathways can cleave complement component 3 (C3) into two fragments, namely C3a, a pro-inflammatory factor, and C3b, which shows opsonization and contributes to the formation of C3 and complement component 5 convertases [8]. C3 is the central component of the complement system, and the cleavage of C3 is often considered the marker of complement system activation [9,10].

The AP is regarded as the most evolutionary ancient complement activation pathway [11], appearing early in echinoderm such as sea urchins [12,13]. Many components of the AP have been identified in echinoderm sea urchins [12,13], tunicate ascidians [14], cephalochordate amphioxus [15], jawless fish lampreys [16], cartilaginous fish [17], teleost fish [18], amphibians [19], reptiles [20], birds [21], and mammals [22]. In mammals, the activation mechanism of the AP has been clearly defined. Unlike in the CP and LP, the activation of C3 in the AP relies on complement factor B (Bf) [22]. Bf is an important serine protease, and its partial fragment Bb can recruit C3b and form the C3 convertase (C3bBb), which finally promotes the cleavage of C3 and complement system activation [23,24]. Complement factor D (Df), another serine protease in the AP, can hydrolyze Bf at the amino acid site of Arg–Lys and cleave Bf into two fragments of Ba and Bb [25,26]. Therefore, the Df protein is effective in promoting the activation of the AP. In humans, it has been reported that the deficiency of Df weakens the immune responses against *Neisseria meningitidis*, leading to meningitis [27]. In mice, the deficiency of Df brings about the widespread replication of *Escherichia coli* in the intestinal tract and induces colitis [28]. In addition, Df has also been demonstrated to participate in antiviral immunity. During virus infection, Df upregulation enhances the production of the C3bBb complex and promotes C3 cleavage and activation of the AP [8]. Further studies show that Df-enhanced AP activation can lead to the destruction of virus-infected cells via the membrane attack complex of the complement system, which helps the hosts eliminate the invading virus [29]. This evidence indicates that Df plays a vital role in the antibacterial and antiviral immune responses of mammals.

Dfs have been identified in teleost fish, such as *Cyprinus carpio* [30], *Salvelinus fontinalis* [31], *Oncorhynchus mykiss* [32], *Paralichthys olivaceus* [33], *Ictalurus punctatus* [34], *Oryzias latipes* [35], *Oplegnathus fasciatus* [36], *Megalobrama amblycephala* [37], and *Carassius auratus* [38], and structural information and the mRNA expression profiles during pathogen infection were revealed in these studies. Compared to Dfs in mammals, the structure of Dfs in teleost species is highly conserved, with a Tryp_SPc domain, three substrate binding sites, and three catalytic sites [25,39]. It has been reported that the mRNA expression level of Df in various teleost species is significantly changed after pathogen infection. For example, Df transcript levels are significantly increased in the kidney, liver, and spleen of *P. olivaceus* after viral hemorrhagic septicemia virus (VHSV) infection [33]. The expression profiles of Df in *O. fasciatus* show significant upregulation at 6 and 12 h after rock bream iridovirus (RBIV) infection [36]. After *Aeromonas hydrophila* challenge, the expression of Df in *M. amblycephala* was also upregulated 3.7-fold and 16-fold in the liver and kidney, respectively [37]. This evidence strongly suggests the involvement of Df in the immune defense against bacteria and viruses in teleost species. However, the functional mechanisms of Df in the AP and in withstanding pathogen infection are far from being clarified in these species.

Grass carp, *Ctenopharyngodon idella*, is a very important freshwater teleost fish cultured in China, and its production reached 5.53 million tons in 2019 [40]. However, grass carp aquaculture is severely restricted by grass carp hemorrhagic disease, which is caused by a double-stranded RNA virus known as grass carp reovirus (GCRV) [41,42,43,44]. In order to better control grass carp hemorrhagic disease, the molecular basis of grass carp resistance to GCRV should be urgently investigated. Based on transcriptome analysis in *C. idella*, we have previously found that the mRNA expression of Df (designated as *Ci*Df) was significantly upregulated 2.18-fold at 72 h after GCRV infection, suggesting the involvement of *Ci*Df in immune defense against GCRV infection [45]. In the present study, we cloned the full-length *Ci*Df cDNA sequence and explored its functions. The aims of this study were to (1) analyze the structural and evolutionary characteristics of *Ci*Df, (2) verify whether its function in the AP is conserved, and (3) reveal its potential functional mechanism after GCRV infection, which can provide a fundamental basis for studying the roles of Df and the AP in grass carp during GCRV infection.

## 2. Results

### 2.1. Sequence Characteristics of CiDf

**Figure 1:**
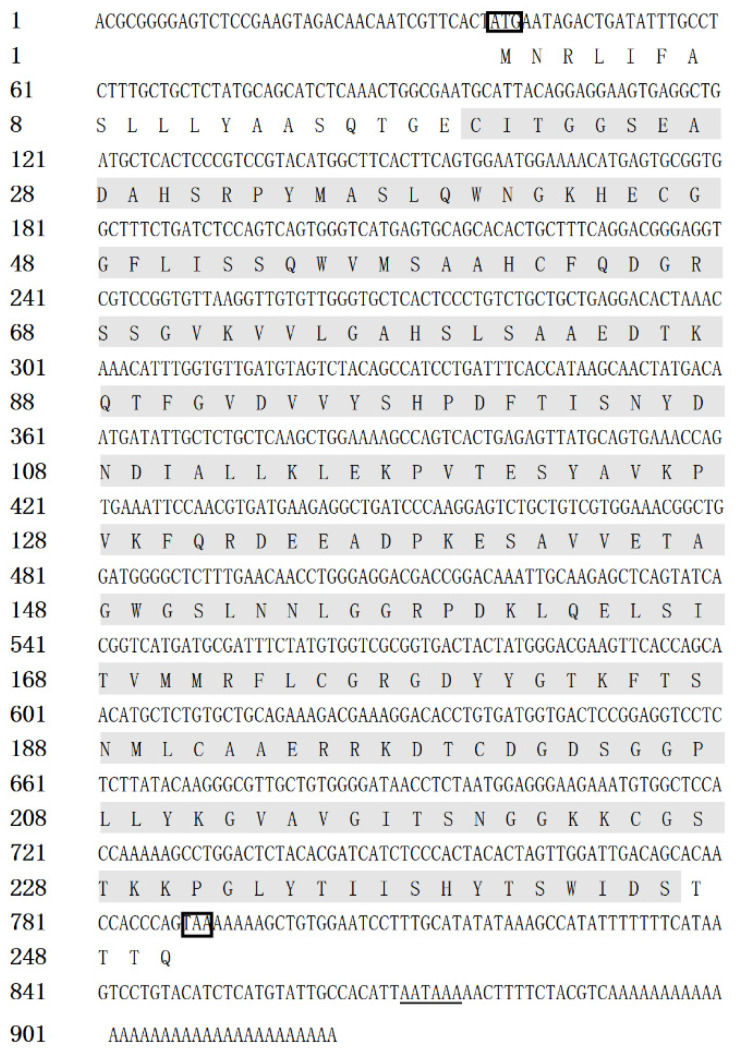
The full-length cDNA and amino acid sequence of *Ci*Df. The single Tryp_SPc domain (20–244 aa) is marked with a gray shadow, the initiation codon and stop codon are marked with a black box, and the “AATAAA” mRNA tail is underlined.

The full-length cDNA sequence of *Ci*Df (GenBank accession number: KF672346.1) is 922 base pairs (bp), with a 38 bp 5′ terminal untranslated region (UTR), an open reading frame (ORF) of 753 bp that encodes 250 amino acids, and a 131 bp 3′ UTR containing an “AATAAA” mRNA tail (Figure 1). Based on prediction using the Compute pI/Mw tool in ExPASy, the molecular weight of the *Ci*Df protein is about 27.06 kDa, with a deduced theoretical isoelectric point of 6.30.

**Figure 2:**
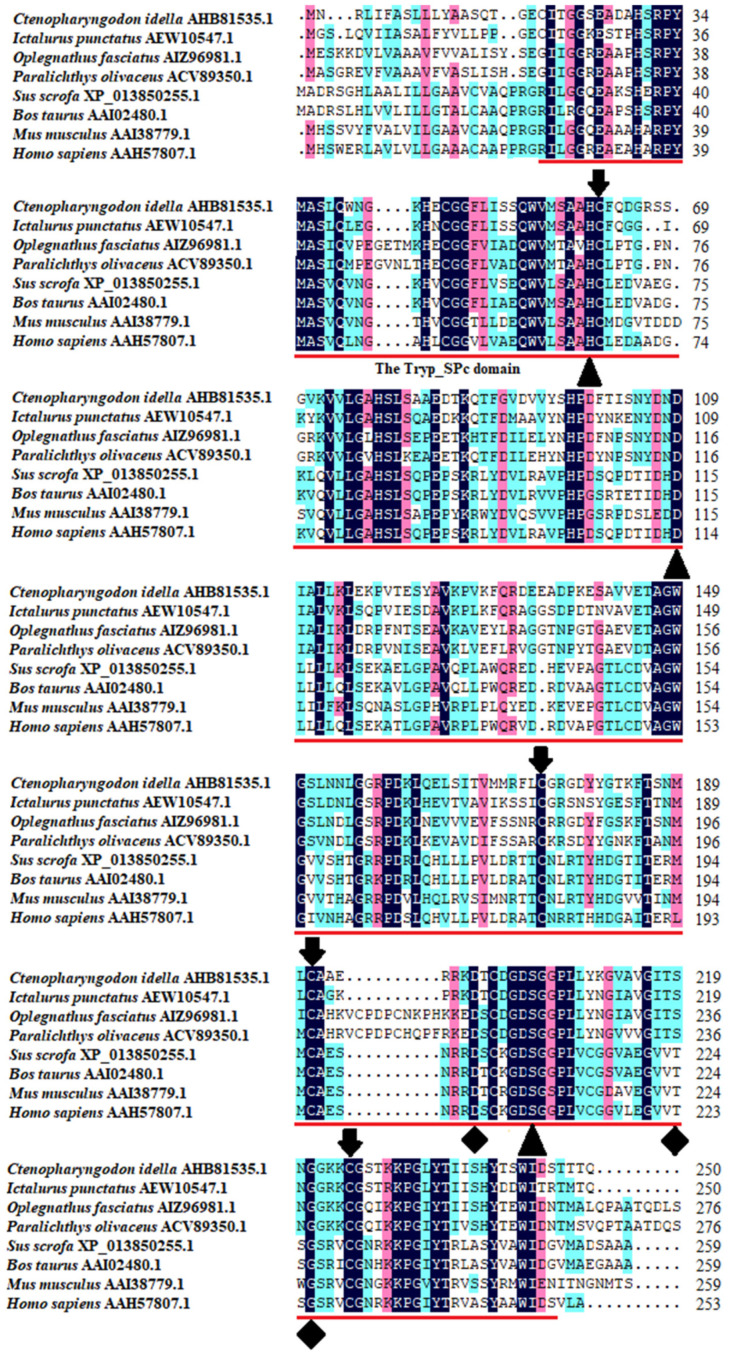
Multiple sequence alignments of *Ci*Df with other vertebrates. The amino acid residues shaded in dark are conserved sites, and the functional domain is underlined in red. The critical residues of catalytic sites (serine protease active sites), the substrate-binding sites, and residues probably involved in disulfide pairing are indicated as solid triangles, solid diamonds, and solid arrows, respectively.

**Figure 3:**
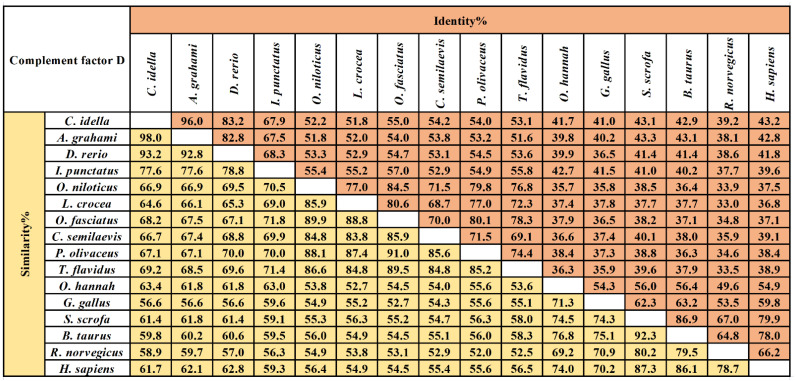
The amino acid sequence identity and similarity of Dfs among different vertebrates. The selected Dfs were from vertebrates including *Ctenopharyngodon idella* (AHB81535.1), *Anabarilius grahami* (ROK15838.1), *Danio rerio* (NP001018368.1), *Ictalurus punctatus* (AEW10547.1), *Oreochromis niloticus* (XP003447819.1), *Larimichthys crocea* (KKF28223.1), *Oplegnathus fasciatus* (AIZ96981.1), *Cynoglossus semilaevis* (XP008314450.1), *Paralichthys olivaceus* (ACV89350.1), *Takifugu flavidus* (TWW72489.1), *Channa striata* (SSC14279.1), *Gallus* *gallus* (XP_040548688.1), *Sus scrofa* (XP_013850255.1), *Bos taurus* (NP001029427.1), *Rattus norvegicus* (NP001071110.1), and *Homo sapiens* (NP001919.2). The values of similarity and identity are backgrounded in yellow and orange respectively.

The multiple sequence alignment results showed that three residues for substrate binding (^198^Asp, ^219^Ser, and ^221^Gly), three residues of the catalytic triad (^61^His, ^109^Asp, and ^204^Ser), and four cysteine residues probably involved in disulfide pairing (^62^Cys, ^175^Cys, ^191^Cys, and ^225^Cys) were all conserved in *Ci*Df (Figure 2). The pairwise alignment analyses revealed that the deduced amino acid sequence of *Ci*Df shared 52.0–98.0% similarity and 33.0–96.0% identity with those of the known Dfs and exhibited the highest similarity (98%) and identity (96%) with Df from *Anabarilius grahami* (Figure 3).

### 2.2. The Domain Architecture and Three-Dimensional Structure Model of CiDf

**Figure 4:**
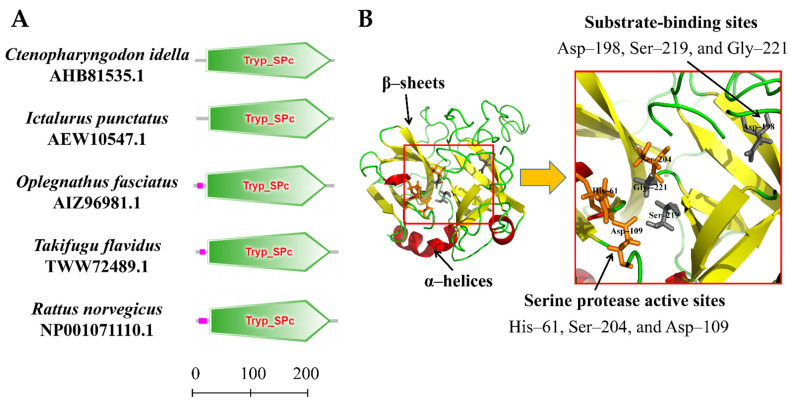
Protein domains and three-dimensional structure of *Ci*Df. (**A**) Protein domains of Dfs from *Ctenopharyngodon idella*, *Ictalurus punctatus*, *Oplegnathus fasciatus*, *Takifugu flavidus*, and *Rattus norvegicus* were compared. Tryp_SPc represents the trypsin-like serine protease. The scale on the bottom margin indicates amino acid number. (**B**) In the three-dimensional structure of *Ci*Df, the catalytic sites are colored in orange, and the substrate-binding sites in gray.

The amino acid sequence of the *Ci*Df protein was input into the online software SMART for the prediction of protein domain architecture. The results showed that *Ci*Df contained a highly conserved Tryp_SPc domain ranging from 20 to 244 amino acids, similar to Dfs from *I. punctatus*, *O. fasciatus*, *Takifugu flavidus*, and *Rattus norvegicus* (Figure 4A). Three-dimensional homology modeling of the *Ci*Df protein was conducted using I-TASSER based on human Df (GenBank accession number: NP001919.2) as the template, and the results reveal that the *Ci*Df protein consists of 3 α-helices and 13 β-sheets, three conserved residues (^61^His, ^109^Asp, and ^204^Ser) as the catalytic triad, and three conserved binding sites (^198^Asp, ^219^Ser, and ^221^Gly) in the substrate specificity pocket (Figure 4B).

### 2.3. The Phylogenetic Tree

**Figure 5:**
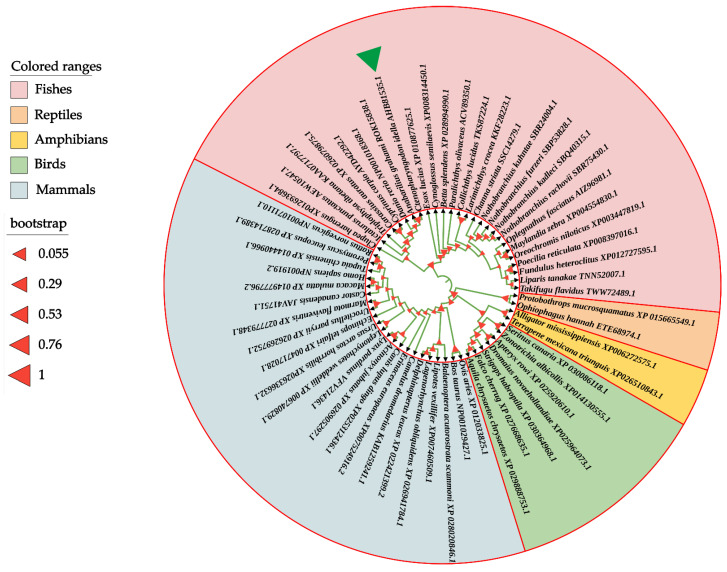
Phylogenetic relationship of Df proteins from various vertebrates. The phylogenetic tree is constructed using the neighbor-joining method and MEGA 6.06 software based on full-length Df protein sequences from different species. The *Ci*Df protein is indicated by a green solid triangle. All of the selected Df proteins are separated into five branches that are marked by different colors.

To determine the evolutionary feature of the *Ci*Df protein, a phylogenetic tree was constructed based on full-length Df amino acid sequences from various vertebrates. The results showed that Dfs from mammalians, birds, reptiles, amphibians, and fishes were obviously separated into five branches in the phylogenetic tree. The *Ci*Df was firstly clustered with that of *A. grahami* (ROK15838.1) and then clustered with Cyprinidae Dfs of *Danio rerio* (NP001018368.1), *C. carpio* (AYD42292.1), and *C. auratus* (XP_026079875.1). Finally, these five Dfs together with 21 other Dfs, mainly from Sciaenidae, Cichlidae, and Clupeidae species, formed a distinct fish branch (Figure 5).

### 2.4. The Analysis and Comparison of CiDf Genomic Structure

**Figure 6:**
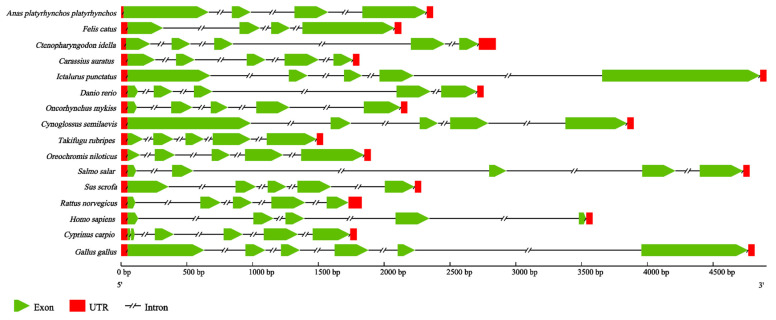
Genomic structure arrangements of Dfs from grass carp and other vertebrates. The red boxes, green wedges, and broken lines represent UTRs, exons, and introns of Dfs, respectively. All of the genomic sequences, including Dfs from *Anas platyrhynchos platyrhynchos* (ENSAPLG00000023562), *Felis catus* (ENSFCAG00000007308), *Carassius auratus* (ENSCARG00000016075), *Ictalurus punctatus* (ENSIPUG00000006895), *Danio rerio* (ENSDARG00000039579), *Oncorhynchus mykiss* (ENSOMYG00000016711), *Cynoglossus semilaevis* (ENSCSEG00000015236), *Takifugu rubripes* (ENSTRUG00000028684), *Oreochromis niloticus* (ENSONIG00000029006), *Salmo salar* (ENSSSAG00000064487), *Sus scrofa* (ENSSSCG00060010731), *Rattus norvegicus* (ENSRNOG00000033564), *Homo sapiens* (ENSG00000197766), *Cyprinus carpio* (ENSCCRG00000029196), and *Gallus gallus* (ENSGALG00000040832), were obtained from the ensemble database.

In order to identify features of the *Ci*Df genomic structure, we constructed the genomic structure schematic arrangements of *Ci*Df and its counterparts from 15 other vertebrates. The results revealed that the genomic sequence of *Ci*Df (2451 bp) comprised five exons interrupted by four introns. Although the full-length genomic DNA sequences of *Ci*Df and its counterparts from other vertebrates obviously varied, *Ci*Df possessed the same number of exons and introns as those from most vertebrates, except for the Dfs from duck (*Anas platyrhynchos*) (four exons and three introns), cat (*Felis catus*) (four exons and three introns), common carp (*C. carpio*) (six exons and five introns), and chicken (*Gallus gallus*) (six exons and five introns) (Figure 6).

### 2.5. The Enhancement of rCiDf to Cleave C3 Protein in Grass Carp Serum

**Figure 7:**
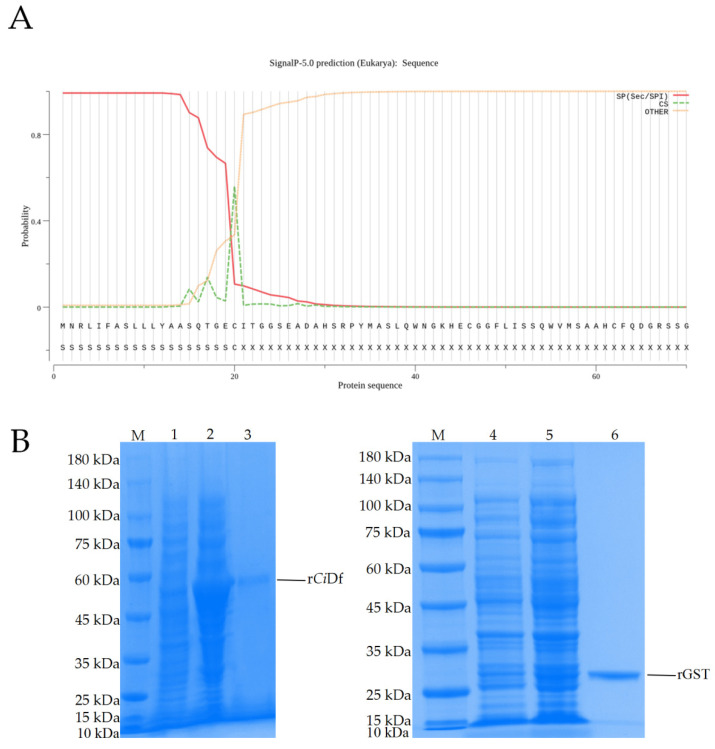
Signal peptide prediction for the *Ci*Df protein and SDS-PAGE analysis of the recombinant *Ci*Df protein. (**A**) The first 20 amino acid residues were predicted to form a signal peptide in the *Ci*Df protein using the SignalP-5.0 tool. (**B**) SDS-PAGE analysis of recombinant *Ci*Df protein. M: marker; Lane 1: lysates of pGEX-4T-1-*Ci*Df plasmid transformed bacteria before induction; Lane 2: lysates of pGEX-4T-1-*Ci*Df plasmid transformed bacteria after induction with 1 mM IPTG; Lane 3: purified r*Ci*Df protein; Lane 4: lysates of pGEX-4T-1 plasmid transformed bacteria before induction; Lane 5: lysates of pGEX-4T-1 plasmid transformed bacteria after induction with 1 mM IPTG; Lane 6: purified rGST protein.

Prediction using the SignalP-5.0 tool revealed the residues of ^1^Met~^20^Cys for a signal peptide in *Ci*Df protein (with the probability of 0.5595, Figure 7A). To study the function of the *Ci*Df protein in vitro, a recombinant *Ci*Df protein (r*Ci*Df) of the mature peptide fragment of *Ci*Df (^21^Ile~^250^Gln) with a GST-tag was constructed, expressed, and then purified using a GST resin column. As shown in SDS-PAGE, a main band of r*Ci*Df with a molecular mass of about 52 kDa was observed in Lane 3, which corresponded approximately with the predicted molecular mass of *Ci*Df fused to a GST-tag (Figure 7B).

**Figure 8:**
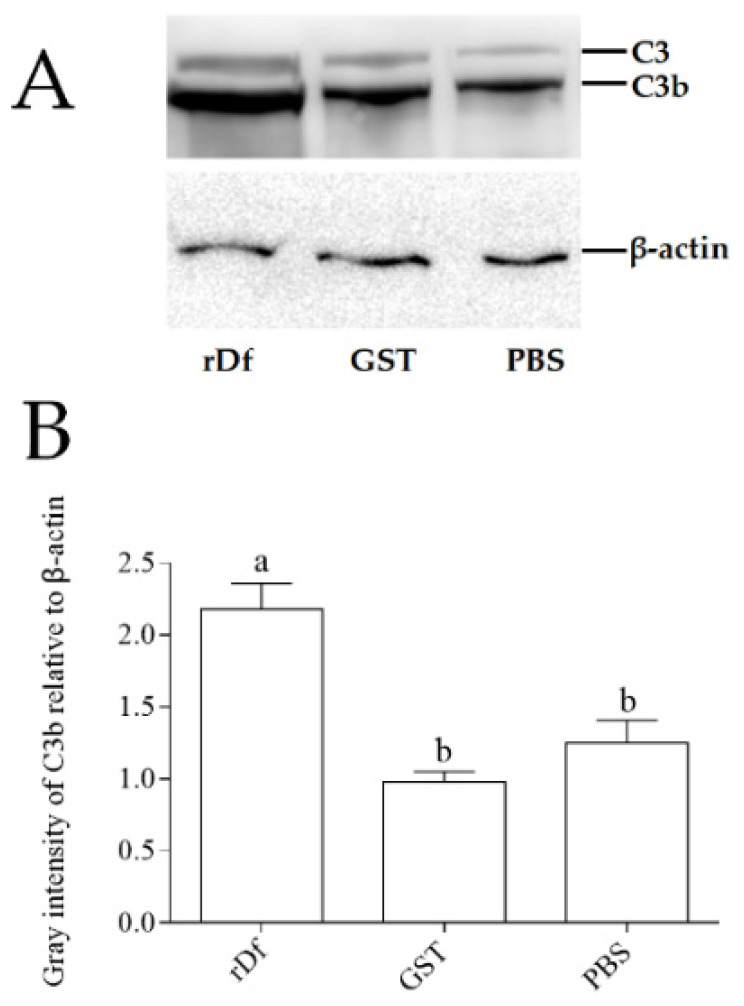
r*Ci*Df activity promotes C3 cleavage in grass carp serum. (**A**) Western blotting analysis of C3 protein cleavage. Bands of C3 and C3b protein were imaged via the GeneSys Imaging System. β-actin was used as internal reference protein. (**B**) Gray intensity analysis of the C3b protein in grass carp serum using ImageJ. The letters a and b indicate significant differences among groups (*p* < 0.05).

It has been demonstrated that Df can promote the cleavage of C3 into C3a and C3b. In order to verify whether the function of the *Ci*Df protein was conserved, the purified r*Ci*Df protein was incubated with grass carp serum overnight, and western blot analysis was conducted using the rabbit-anti-grass-carp C3 polyclonal antibody for the detection of C3 cleavage in grass carp serum. rGST proteins and PBS incubated with grass carp serum were set as the negative and blank control group, respectively. The results of the r*Ci*Df group (rDf), the blank control group (PBS), and the negative control group (GST) all revealed two distinct bands of C3 and C3b in grass carp serum (Figure 8A). In addition, gray intensity analysis with β-actin as an internal reference protein showed that the relative content of the C3b protein in grass carp serum incubated with r*Ci*Df was significantly higher than those in the negative control group (2.22-fold, *p* < 0.05) and the blank control group (1.74-fold, *p* < 0.05) (Figure 8B).

### 2.6. The Distribution of CiDf mRNA Transcript in Grass Carp Tissues

**Figure 9:**
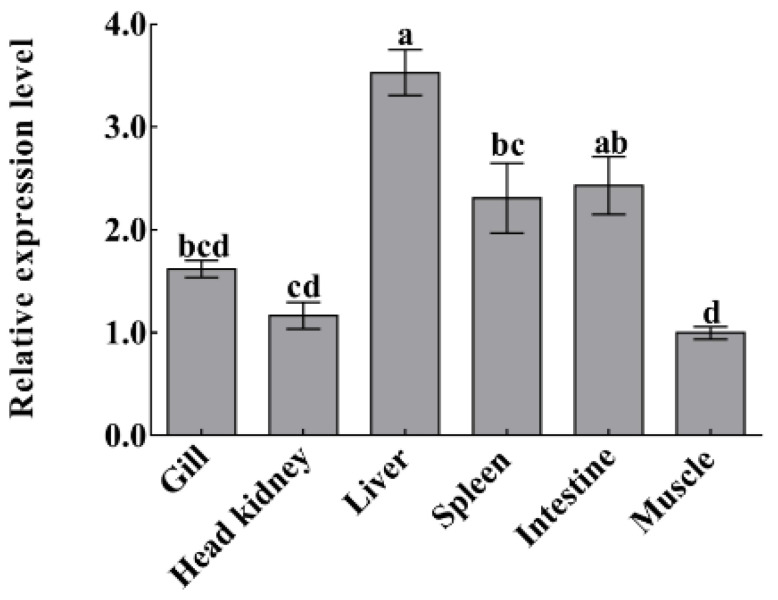
The mRNA expressions of *Ci*Df in various tissues from uninfected grass carp. Vertical bars represent the mean ± SD. The letters a–d represent significant differences among tissues (*p* < 0.05).

Quantitative real-time polymerase chain reaction (*q*PCR) was carried out to detect the mRNA expression level of *Ci*Df in different tissues of uninfected grass carp, including the gill, head kidney, liver, spleen, intestine, and muscle. There was only one peak at the corresponding melting temperature for *Ci*Df in the dissociation curves (data not shown). *Ci*Df transcripts were most abundant in the liver, where their levels were 3.53-fold higher than in muscle (*p* < 0.05). The expression levels in the intestine and spleen were also relatively high—2.43- and 2.31-fold higher than in muscle, respectively (*p* < 0.05). The lower expressions were observed in the gill and head kidney—1.62- and 1.17-fold lower than in muscle, respectively (Figure 9).

### 2.7. The Fold Changes in CiDf mRNA Expression during GCRV Infection

**Figure 10:**
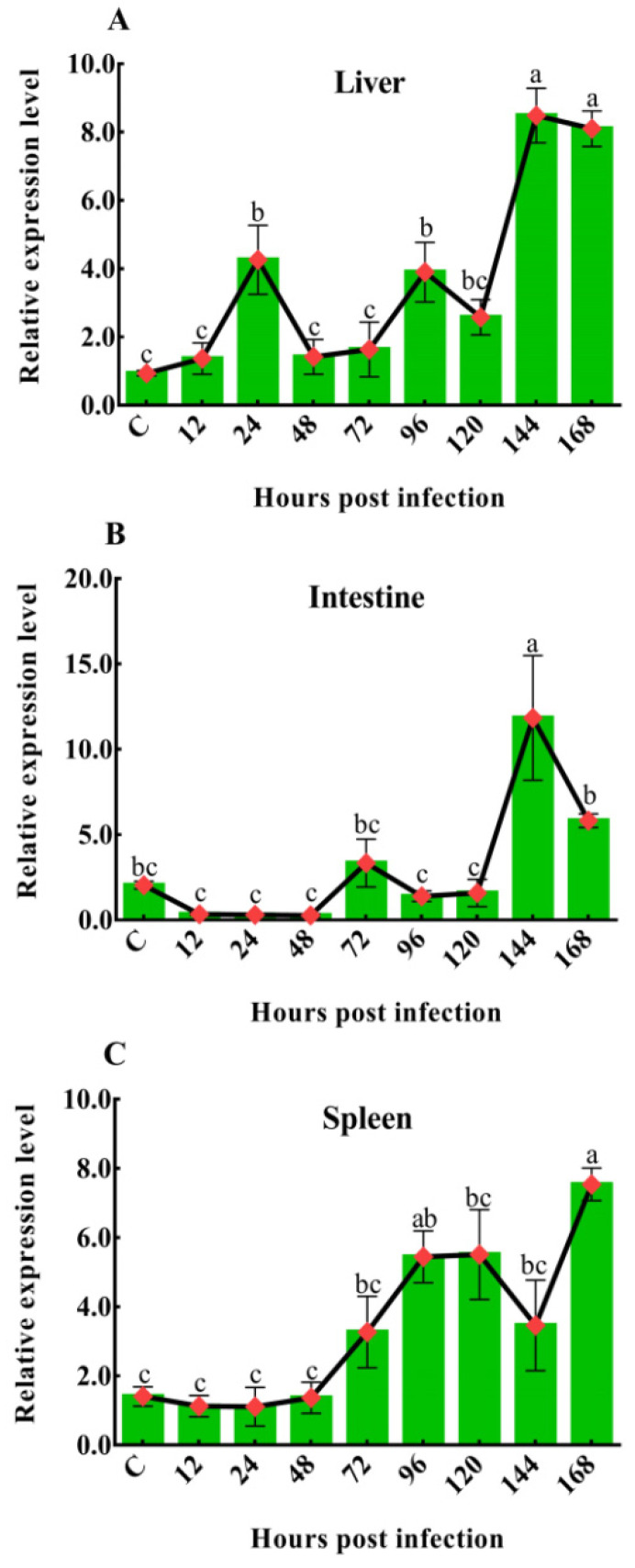
The mRNA expression patterns of *Ci*Df in the liver (**A**), intestine (**B**), and spleen (**C**) during GCRV infection. Vertical bars represent the mean ± SD. The letters a–c represent significant differences among the expressions in the liver, spleen, and intestine at various time points post-GCRV challenge (*p* < 0.05).

To investigate the dynamic changes in *Ci*Df during GCRV infection, its mRNA expression levels in the liver, spleen, and intestine of grass carps after GCRV challenge at 12, 24, 48, 72, 96, 120, 144, and 168 h were characterized by *q*PCR. In the liver, the mRNA expression level of *Ci*Df gradually increased and then decreased at the time periods of 12–48 h and 48–120 h, while it was sharply upregulated at 120 h after the GCRV challenge, reached a maximum at 144 h, at which point its levels were significantly higher (*p* < 0.05) than at other time points, except 168 h (Figure 10A). In the intestine, there was no extremely significant change in *Ci*Df mRNA expression at 12–120 h post-GCRV challenge, while the mRNA expression level of *Ci*Df sharply increased and reached a maximum at 144 h, and it was significantly higher than at all other time points post-GCRV challenge (*p* < 0.05) (Figure 10B). In the spleen, the expression level of *Ci*Df gradually increased from 12 to 120 h and decreased at 144 h post-GCRV challenge, while it steadily increased and reached a maximum at 168 h (*p* < 0.05) (Figure 10C).

## 3. Discussion

The complement system is the humoral backbone of innate immune defense, and it is a link between innate and adaptive immune responses, which comprise. More than 35 distinct plasma and membrane-bound proteins, forming three convergent pathways of activation: the CP, LP, and AP [46,47]. An activated complement system marked by the cleavage of C3 plays multiple immune roles, including in the elimination of invading pathogens [48], promotion of inflammatory response [49,50], and clearance of apoptotic cell and necrotic cell debris [51,52], in addition to the modulation of adaptive immunity [53,54,55]. From an evolutionary perspective, the AP is regarded as the most ancient complement activation pathway, appearing early in echinoderms [11]. The AP can be triggered by the direct recognition of certain microbial surface structures [7,56], and AP-regulating proteins, including Bf [57], Df [58], and complement P factor [59], tightly control this pathway. Df, a member of the chymotrypsin family of serine proteases, plays a pivotal role in both initiation and amplification loops of complement system activation by continuously promoting C3 cleavage in the AP [60,61]. It has been reported in many human and mouse disease models that the deficiency or dysfunction of Df weakens the host immune killing ability to foreign pathogens, including viruses and bacteria [8,27,28,29,62,63]. There is also substantial evidence that Df mRNA expression is upregulated after pathogen infection in various fish species such as *O. fasciatus* [36], *M. amblycephala* [37], and *C. auratus* [38], suggesting its importance in fish immune defense. In our previous study, the transcriptome data show that *Ci*Df mRNA expression is significantly upregulated 2.18-fold after GCRV infection in *C. idella* [45], so we infer that *Ci*Df is involved in the immune defense during GCRV infection. In the present study, we cloned the full-length cDNA of *Ci*Df, ascertained its structure and functional characteristics, investigated its expression patterns after GCRV infection, and attempted to further reveal its potential mechanism in defense against GCRV infection.

It has been confirmed that Df is structurally different from other serine proteases in the complement system and only contains a single serine protease superfamily domain, called the Tryp_SPc domain, which can cleave Bf into Ba and Bb [25,26]. In addition, Df is unique among serine proteases in that it requires neither enzymatic cleavage for the expression of proteolytic activity nor inactivation by a serpin for its control [64]. To understand this unique functional characteristic, the crystal structure of human Df was resolved, and two features essential for catalysis are shown within the spatial structure of Df: (1) a catalytic triad and (2) a substrate specificity pocket [65,66]. The crystal structure data suggest that the regulation of Df activity may be achieved by a novel mechanism that depends on reversible conformational changes for the expression and control of catalytic activity [65,66]. These conformational changes are believed to be induced by a single natural substrate, Bf, and result in the realignment of the catalytic triad and the specificity pocket [67,68,69]. The importance of Asp, His, and Ser, the three residues that form the “catalytic triad” of human Df, has recently been established by kinetic analyses and chemical modification experiments [68]. The essential function of these three residues in the substrate specificity pocket of human Df has also been verified by site-directed mutagenesis [70]. In the present study, the results of amino acid sequence similarity and phylogenetic tree analyses show that *Ci*Df shares an amino acid sequence similarity of 52.0–98.0% with Dfs from the selected vertebrates and that it is evolutionarily divergent from the Dfs of mammalians, birds, reptiles, and amphibians. Nevertheless, we find that the *Ci*Df also harbors a single Tryp_SPc domain (^20^Cys~^244^Ser) with three conserved residues, ^61^His, ^109^Asp, and ^204^Ser, as the catalytic triad, and three conserved binding sites, ^198^Asp, ^219^Ser, and ^221^Gly, in the substrate specificity pocket, similarly to the Dfs reported from mammalians. Therefore, the results show that *Ci*Df possesses the key residues for catalysis and substrate binding, suggesting its functions in the complement system are conserved in grass carp.

In mammals, the function of Df in the AP is relatively clear. Df catalyzes the hydrolysis of the single Arg–Lys bond of Bf—the only known natural substrate of Df in the AP—into Ba and Bb fragments and forms a complex with Bb and C3b that is an alternative C3 convertase [25,26,71,72,73]. Subsequently, activity of this C3 convertase leads to the cleavage of C3 and the activation of terminal complement components and to the formation of a membrane attack complex that eliminates invading microbes or host cells infected with viruses [10,74]. In addition, Df is a component absolutely required for the AP, since it is the only enzyme in mammalian blood able to catalyze C3bBb formation [61,62,75]. Therefore, Df plays an important role in the complement system against invading pathogens and is considered a vital target for the pharmaceutical control of complement activation. Fish possess complement systems similar to those in mammals, and the identified fish complement proteins have many similarities to their mammalian counterparts. However, few studies have been conducted on teleost fish Df, particularly with regard to its functional aspects and regulation after pathogen infection. The sequence properties of Dfs from *C. carpio* [30] and *P. olivaceus* [33] as well as the sequence properties and expression analysis of Dfs in *I. punctatus* [34] and *S. fontinalis* [31] have been reported. The maternal transfer of the Df protein to offspring has also been confirmed in *O. mykiss* [32]. Although the protease activity of the Df protein has been detected in *O. fasciatus* [36], the activities of teleost Dfs in relation to functions in the AP remain unverified. In the present study, in vitro experiments reveal that the incubation of r*Ci*Df significantly increases the C3b protein content in grass carp serum, directly demonstrating the functional conservation of *Ci*Df in the AP, which can promote the cleavage and activation of C3 in the grass carp complement system, similarly to Dfs in mammals.

The expression profiles of immune molecules during pathogen infection may reflect the process of host–pathogen interaction. Substantial data have shown quite different expression patterns for Dfs from various fish. For example, the mRNA transcripts of Df are mainly distributed in the liver of *C. auratus*, and few are distributed in the blood [38], while the mRNA transcripts of Dfs from *M. amblycephala* [37] and *I. punctatus* [34] are most abundant in the kidney and spleen, respectively. Our results in the present study show that the mRNA expression of *Ci*Df is highest in the liver and lowest in the muscle of grass carp. These observations indicate differences in the main sites where Df mRNAs are synthesized and where proteins exert immune functions from various fish species, which probably contributes to the complex host complement system–pathogen interaction in fish. Investigating the dynamic changes in Df expression during pathogen infection may be helpful for understanding AP activity in host defense. Evidence in teleost fish reveals that the expression of Df mRNA is significantly upregulated after pathogen infection and responds to various pathogens with different patterns. During VHSV infection within 24 h in *P. olivaceus* [33], the expression of Df mRNA in the kidney, liver, and spleen is first upregulated and then downregulated in all cases, peaking more sharply in the kidney at 6 h post-VHSV challenge than in the liver and spleen. In addition, bacterial infection seems to arouse more rapid AP activity than VHSV infection, as observed in *P. olivaceus*, since Df mRNA expression in the kidney and spleen peak significantly at 1 h after the *Streptococcus iniae* challenge, representing an early stage of infection. In *O. fasciatus*, the AP activity appears to be relatively intense in the middle stage of pathogen infection because the Df mRNA expression peaks at 6 or 12 h after the challenge of *Streptococcus iniae* and RBIV when assayed over a 24 h period [36]. In the present study, we attempted to characterize the levels of *Ci*Df mRNA expression in the liver, spleen, and intestine after the GCRV challenge at 12, 24, 48, 72, 96, 120, 144, and 168 h by *q*PCR in order to further investigate the dynamic activities of AP during GCRV infection. The results reveal the maintenance of a high level of *Ci*Df mRNA expression in the liver, spleen, and intestine of grass carp at 144 and 168 h after GCRV challenge, representing the late stage of GCRV infection in grass carp. Because of the important roles of Df in the AP, our expression data collectively indicate that the AP activity still exerts intense defense effects at the late stage of GCRV infection in grass carp. These findings offer useful information for understanding the interaction between the host complement system and GCRV infection in grass carp.

In conclusion, this study identifies an AP-regulating protein, *Ci*Df, from *C. idella*. *Ci*Df harbors the conserved structure, which features a single Tryp_SPc domain, three conserved residues as the catalytic triad, and three conserved binding sites in the substrate specificity pocket. *Ci*Df protein can promote the cleavage and activation of C3, similar to what has been reported for mammalian Dfs. The expression of *Ci*Df mRNA was found to be maintained at a high level until the late stage of GCRV infection, suggesting a distinct functional pattern related to its involvement in the immune defense against GCRV infection compared to other teleost species. These results may provide a key basis for studying the roles of Df and AP during GCRV infection in grass carp.

## 4. Materials and Methods

### 4.1. Cloning of CiDf Full-Length cDNA by Using Rapid Amplification of cDNA Ends (RACE) Technique

An assembled unigene (Unigene5167) of 657 bp annotated as Df from our previous transcriptome data [45] was selected for further cloning of the full-length cDNA of *Ci*Df. Two specific primers, Df 5′ and Df 3′ (Table 1), were designed to amplify the full-length cDNA of *Ci*Df by RACE technology. The total volume of the PCR was 50 µL, including 1 µL of cDNA, 25 µL of 2× EasyTaq PCR SuperMix (Vazyme, Nanjing, China), 1 µL of each gene-specific primer Df 5′ (Df 3′), 1 µL of Universal Primer A Mix, and 22 µL of H_2_O. The PCR programs were run as follows: 5 cycles at 94 °C for 30 s, 72 °C for 3 min; 5 cycles at 94 °C for 30 s, 70 °C for 30 s, and 72 °C for 3 min; 25 cycles at 94 °C for 30 s, 68 °C for 30 s, and 72 °C for 3 min. The PCR products were gel-purified using the MiniBest Agrose Gel DNA Extraction Kit Ver. 4.0 (Takara, Kyoto, Japan), cloned into the pUCm-T vector (Kanglang, Shanghai, China), and then sequenced by M13F(-47) and RV-M primers (Table 1).

### 4.2. Sequence Analysis of CiDf

The open-reading frames and deduced amino acid sequences of *Ci*Df were predicted using the online ExPASy Translate tool (http://web.expasy.org/translate/, accessed on 9 September 2019). The molecular weight and theoretical isoelectric point were calculated using the ExPASy Compute pI/Mw tool (http://web.expasy.org/compute_pi/, accessed on 9 September 2019). The domain architecture of the *Ci*Df protein was predicted using the Simple Modular Architecture Research Tool (SMART) (http://smart.embl-heidelberg.de/, accessed on 9 September 2019). The signal peptide was predicted using the SignalP-5.0 tool (https://services.healthtech.dtu.dk/service.php?SignalP-5.0, accessed on 31 October 2019). Multiple amino acid sequence alignment of Dfs from different species including *C. idella* (AHB81535.1), *I. punctatus* (AEW10547.1), *O. fasciatus* (AIZ96981.1), *P. olivaceus* (ACV89350.1), *S. scrofa* (XP_013850255.1), *B. taurus* (AAI02480.1), *M. musculus* (AAI38779.1), and *H. sapiens* (AAH57807.1) was performed using DNAman software 8.0. A comparative homology analysis was performed using the MatGat program 2.03 to assess the identity and similarity of *Ci*Df with other Dfs. A phylogenetic tree was constructed using the neighbor-joining method and MEGA 6.06 software based on full-length Df protein sequences from different species (information shown in Table 2). A bootstrapping test was adopted with 1000 replications, and the phylogenetic tree was then edited online using the iTOL tool (http://itol.embl.de, accessed on 2 September 2020). The entire cDNA and genomic DNA sequences of *Ci*Df were aligned together to determine the exon–intron organization using the Spidey genomic alignment tool (http://www.ncbi.nlm.nih.gov/spidey/, accessed on 15 August 2021) available at NCBI. Other genomic structures of Dfs used for comparison were obtained through the Ensemble Genome Browser database (http://asia.ensembl.org/index.html, accessed on 15 August 2021). Respective genomic arrangements were viewed using the Gene Structure Display Server (http://gsds.gao-lab.org/, accessed on 15 August 2021).

### 4.3. Prokaryotic Expression, Purification of Recombinant CiDf Protein

A pair of specific primers (rDf F and rDf R, Table 1) were designed to amplify the cDNA sequence encoding the mature peptide of *Ci*Df (^21^Ile~^250^Gln) with *BamH* I and *EcoR* I cleavage site sequences added to the 5′ end. The PCR fragment was digested using the *BamH* I and *EcoR* I restriction enzymes (New England Biolabs, Ipswich, UK) and ligated into the expression vector pGEX-4T-1 (Vazyme, Nanjing, China). The recombinant plasmid of pGEX-4T-1-*Ci*Df was transformed into *Escherichia coli* BL21(DE3) and cultured overnight. The positive transformants were picked and incubated in LB medium containing 100 μg/mL ampicillin (Beyotime, Shanghai, China) at 37 °C via shaking at 220 rpm for 4 h. When the OD_600_ reached 0.4–0.6, isopropyl-β-d-thiogalactopyranoside (IPTG) (Beyotime, Shanghai, China) was added to the LB medium at a final concentration of 1 mM and incubated at 16 °C via shaking at 140 rpm. After inducible expression for 24 h, the bacterial culture was sonicated and centrifuged to obtain the supernatant. The supernatant was further filtered using a 0.45 μm filter membrane for protein purification. r*Ci*Df was purified using a GST resin column and dialyzed against PBS buffer at 4 °C for 24 h. The protein was separated by reducing 12% sodium dodecyl sulfate-polyacrylamide gel electrophoresis (SDS-PAGE) (GenScript, Nanjing, China) and visualized with Coomassie Bright Blue R250 (Bio-Rad, Hercules, CA, USA). The concentration of purified r*Ci*Df was quantified using the BCA method. The obtained r*Ci*Df was stored at −80 °C for subsequent experiments.

### 4.4. The Incubation of rCiDf with Grass Carp Serum

The activity of r*Ci*Df to promote the cleavage of C3 protein was analyzed according to previous descriptions [23]. Briefly, 25 µL of r*Ci*Df protein (40 µg/mL) were incubated with the serum of grass carp at 16 °C overnight. Equivalent rGST (40 µg/mL) proteins and PBS incubated with grass carp serum were set as the negative and blank control, respectively. Western blot analysis was conducted for the detection of C3 protein cleavage in grass carp serum. These three sample types were detected by first separating proteins using 10% SDS-PAGE followed by transfer to a polyvinylidene difluoride membrane (Millipore, MA, USA). The membranes were blocked in a QuickBlock Blocking Buffer (Beyotime, Shanghai, China) for 15 min at 25 °C and incubated with the rabbit-anti-grass-carp C3 polyclonal antibody (diluted by 1:1000) overnight. After washing three times with Tris-buffered saline containing 0.05% Tween-20, the membrane was incubated with a 1:2000 dilution of HRP AffiniPure goat anti-rabbit IgG (Abclonal, Wuhan, China) for 1 h at 25 °C. The membranes were finally incubated in the BeyoECL Plus substrate system (Beyotime, Shanghai, China) for imaging under the GeneSys Imaging System (Alcatel, Paris, France). The band intensity was quantified and analyzed using ImageJ software. The β-actin was used as an internal reference protein.

### 4.5. Grass Carps, the GCRV Challenge Experiment, and Sampling

Grass carps (*C. idella*, 10~15 cm in body length) were collected from the Institute of Fisheries Science in Xiangyin County, Hunan Province, China. The fish were acclimated for one week in recirculating freshwater tanks at 28 °C and fed a commercial diet according to 3% of their body weight twice a day before processing. The animal experiments were according to the rules of the Animal Care and Use Committee of Hunan Agricultural University (Changsha, China; Approval Code: 201903295; Approval Date: 13 September 2019).

A total of 80 grass carps were employed for the GCRV challenge experiment, and they were randomly divided into two groups. One group was intraperitoneally injected with 200 µL of GCRV 918 (0.2 µL/g body weight, kindly provided by professor Zeng Lingbing from the Yangtze River Fisheries Research Institute of the Chinese Academy of Fishery Sciences) and set as the experimental group. The other group was intraperitoneally injected with the equivalent volume of PBS buffer for use as the control group. The grass carps were randomly sampled (five individuals per time point) at 12, 24, 48, 72, 96, 120, 144, and 168 h post-GCRV challenge in the experimental group and the control group. The spleen, intestine, and liver tissues of every individual were collected and stored at −80 °C before RNA extraction.

### 4.6. Quantitative Real-Time Polymerase Chain Reaction Analysis of CiDf Expression in Different Tissues

For the analysis of gene expression patterns in different tissues, the gill, head kidney, liver, spleen, intestine, and muscle were collected from five uninfected individuals. Total RNA was extracted from these tissues using Total RNA Kit II (Omega, Norcross, GA, USA) following the manufacturer’s instructions. The concentration of RNA was measured with a spectrophotometer (Eppendorf BioSpectrometer basic, Hamburg, Germany), and RNA integrity was evaluated using 1.0% agarose gel electrophoresis. Total RNAs with an OD260/280 value ranging from 1.8 to 2.0 were used for cDNA synthesis. The total RNA samples from various tissues were treated with DNase I and reverse-transcribed into cDNA using a Revert Aid First Strand cDNA Synthesis kit (Thermo Fisher Scientific, Waltham, MA, USA).

*q*PCR was performed on a CFX96 Touch Real-Time PCR Detection System (Bio-Rad, Hercules, California, USA). A pair of specific primers, *Ci*Df YF and *Ci*Df YR (Table 1), were used to amplify a DNA fragment of 92 bp from *Ci*Df. β-actin and 18S RNA genes were employed as internal controls (Table 1). Amplifications were performed in triplicate in a total volume of 10 µL, which contained 5 µL of ChamQ^TM^ Universal SYBR *q*PCR Master Mix (Vazyme, Nanjing, China), 1 µL of diluted cDNA, 0.4 µL of each primer, and 3.2 µL of H_2_O. The cycle conditions were as follows: 1 cycle of 95 °C for 3 min, 40 cycles of 95 °C for 15 s, 60 °C for 15 s, and 72 °C for 15 s. The relative expression levels of genes were analyzed with the Ct method (2^−ΔΔCt^ method) [76].

### 4.7. Temporal Expression Analysis of CiDf in Response to GCRV Infection

Total RNA was isolated from the spleen, intestine, and liver of grass carps in the experimental group and control group at different time points of the GCRV challenge and reverse-transcribed into cDNA. SYBR Green fluorescent *q*PCR was performed as described above to detect the mRNA expression levels of *Ci*Df post-GCRV infection.

### 4.8. Statistical Analysis

All data are indicated as mean ± standard deviation (N = 3 or 6) and were analyzed with Statistical Package for Social Sciences Version 25.0 (SPSS Inc., Chicago, IL, USA). The significant differences among groups were tested by one-way analysis of variance (ANOVA) and multiple comparisons. Differences were considered statistically significant at *p* < 0.05.

## Figures and Tables

**Table 1:** Information regarding primers used in this study.

Primer Name	Primer Sequence (5′-3′)	Application
Df 5′	AGGTTATCCCCACAGCAACGCCCTT	RACE
Df 3′	AGGCTGATGCTCACTCCCGTCCGT	RACE
UPM	CTAATACGACTCACTATAGGGCAAGCAGTGGTATCAACGCAGAGT	RACE
M13F(-47)	CGCCAGGGTTTTCCCAGTCACGAC	Sequence
RV-M	GAGCGGATAACAATTTCACACAGG	Sequence
rDf F	CCGGGATCCATTACAGGAGGAAGTGAG	Prokaryotic expression
rDf R	CCCGAATTCTTACTGGGTGGTTGTGCT	Prokaryotic expression
*Ci*Df YF	ACGACCGGACAAATTGCAAG	*q*PCR
*Ci*Df YR	TGCTGGTGAACTTCGTCCCAT	*q*PCR
β-actin YF	GGCTGTGCTGTCCCTGTATG	*q*PCR
β-actin YR	CTCTGGGCACCTGAACCTCT	*q*PCR
18S RNA YF	ATTTCCGACACGGAGAGG	*q*PCR
18S RNA YR	CATGGGTTTAGGATACGCTC	*q*PCR

Note: F—forward primer; R—reverse primer.

**Table 2:** The information of Dfs from different vertebrates used in phylogenetic analysis.

Species	GenBank Number
*Larimichthys crocea*	KKF28223.1
*Oplegnathus fasciatus*	AIZ96981.1
*Ictalurus punctatus*	AEW10547.1
*Channa striata*	SSC14279.1
*Oreochromis niloticus*	XP003447819.1
*Cynoglossus semilaevis*	XP008314450.1
*Maylandia zebra*	XP004554830.1
*Fundulus heteroclitus*	XP012727595.1
*Clupea harengus*	XP012693684.1
*Danio rerio*	NP001018368.1
*Nothobranchius furzeri*	SBP53828.1
*Ophiophagus hannah*	ETE68974.1
*Terrapene mexicana triunguis*	XP026510843.1
*Ursus arctos horribilis*	XP026336632.1
*Dromaius novaehollandiae*	XP025964073.1
*Canis lupus dingo*	XP025312436.1
*Zonotrichia albicollis*	XP014130555.1
*Poecilia reticulata*	XP008397016.1
*Lipotes vexillifer*	XP007460509.1
*Alligator mississippiensis*	XP006272575.1
*Bos taurus*	NP001029427.1
*Erinaceus europaeus*	XP007524916.2
*Homo sapiens*	NP001919.2
*Rattus norvegicus*	NP001071110.1
*Camelus dromedarius*	KAB1259241.1
*Castor canadensis*	JAV41751.1
*Leptonychotes weddellii*	XP_006740829.1
*Echinops telfairi*	XP_004717028.1
*Delphinapterus leucas*	XP_022421399.2
*Triplophysa tibetana*	KAA0717797.1
*Strigops habroptila*	XP_030364968.1
*Takifugu flavidus*	TWW72489.1
*Serinus canaria*	XP_030086118.1
*Aquila chrysaetos chrysaetos*	XP_029888753.1
*Liparis tanakae*	TNN52007.1
*Protobothrops mucrosquamatus*	XP_015665549.1
*Esox lucius*	XP_010877625.1
*Collichthys lucidus*	TKS87224.1
*Ornithorhynchus anatinus*	XP_028905544.1
*Macaca mulatta*	XP_014977796.2
*Peromyscus leucopus*	XP_028714389.1
*Lynx pardinus*	VFV21436.1
*Balaenoptera acutorostrata scammoni*	XP_028020846.1
*Ovis aries*	XP_012033825.1
*Marmota flaviventris*	XP_027779348.1
*Falco cherrug*	XP_027668635.1
*Tupaia chinensis*	XP_014440966.1
*Penaeus vannamei*	ROT85320.1
*Lagenorhynchus obliquidens*	XP_026941784.1
*Anabarilius grahami*	ROK15838.1
*Acinonyx jubatus*	XP_026905297.1
*Urocitellus parryii*	XP_026269752.1
*Apteryx rowi*	XP_025920610.1
*Nothobranchius kuhntae*	SBR24004.1
*Nothobranchius kadleci*	SBQ40315.1
*Paralichthys olivaceus*	ACV89350.1
*Nothobranchius rachovii*	SBR75430.1
*Cyprinus carpio*	AYD42292.1
*Carassius auratus*	XP_026079875.1

## Data Availability

The data presented in this study are available on request from the corresponding author.
